# Ectopic FOXP3 Expression Preserves Primitive Features Of Human Hematopoietic Stem Cells While Impairing Functional T Cell Differentiation

**DOI:** 10.1038/s41598-017-15689-8

**Published:** 2017-11-17

**Authors:** F. R. Santoni de Sio, L. Passerini, M. M. Valente, F. Russo, L. Naldini, M. G. Roncarolo, R. Bacchetta

**Affiliations:** 1 San Raffaele Telethon Institute for Gene Therapy, IRCCS San Raffaele Scientific Institute, Milan, Italy; 2 San Raffaele Vita-Salute University, Milan, Italy; 3 Department of Pediatrics, Division of Stem Cell Transplantation and Regenerative Medicine, Stanford University School of Medicine, Stanford, CA USA; 4 Institute for Stem Cell Biology and Regenerative Medicine, Stanford University School of Medicine, Stanford, CA USA

## Abstract

FOXP3 is the transcription factor ruling regulatory T cell function and maintenance of peripheral immune tolerance, and mutations in its coding gene causes IPEX autoimmune syndrome. FOXP3 is also a cell-cycle inhibitor and onco-suppressor in different cell types. In this work, we investigate the effect of ectopic FOXP3 expression on HSC differentiation and we challenged this approach as a possible HSC-based gene therapy for IPEX. FOXP3-expressing HSC showed reduced proliferation ability and increased maintenance of primitive markers *in vitro* in both liquid and OP9-ΔL1 co-cultures. When transplanted into immunodeficient mice, FOXP3-expressing HSC showed significantly enhanced engraftment ability. This was due to a pronounced increase in the frequency of repopulating cells, as assessed by extreme limiting dilution assay. Likely underlying the increased repopulating ability, FOXP3 expressing HSC showed significantly enhanced expression of genes controlling stemness features. However, peripheral T cells developed in the FOXP3-humanized mice were quantitatively reduced and hyporesponsive to cytokine and polyclonal stimulation. Our findings reveal unpredicted effects of FOXP3 in the biology of HSC and may provide new tools to manipulate primitive features in HSC for clinical applications. Moreover, they formally prove the need of preserving endogenous FOXP3 regulation for an HSC-based gene therapy approach for IPEX syndrome.

## Introduction

FOXP3 is a forkhead transcription factor controlling the gene expression patterns needed for the function of T regulatory cells (Treg), the main cell subset maintaining peripheral immune tolerance^1^. Highlighting this as its main role, natural mutations in *FOXP3* gene cause the fatal autoimmune *Scurfy* phenotype in mice and the Immune dysregulation, Polyendocrinopathy, Enteropathy, X-linked (IPEX) syndrome in humans, characterized by early-onset severe autoimmunity^2–4^. Although among all cell types the highest FOXP3 expression is detected in Treg cells, several studies have described FOXP3 expression also in human immature thymocyte and T effector cells upon activation^5–7^. In line with this, we have recently demonstrated that alteration of FOXP3 expression leads to intrinsic defects in the development of the T effector cell compartment^8^ (Santoni de Sio *et al*., unpublished data). Moreover, FOXP3 has been described to play different roles beyond the control of peripheral immune tolerance. FOXP3 is a regulator of cell-cycle progression and displays tumor-suppressive functions in both lymphoid and non-lymphoid cells, by controlling the activity of oncogenes such as LMO2, and MYC and SKP2, respectively. Accordingly, loss-of-function of FOXP3 has been associated to hyper-proliferation of normal lymphocytes, T-cell acute lymphoblastic leukemia, prostate and breast carcinomas, and glioblastoma^8–13^.

In light of these findings and in order to challenge different approaches for a hematopoietic stem cell (HSC)-based gene therapy for IPEX syndrome, we addressed here the effect of FOXP3 constitutive expression in the differentiation and homeostasis of HSC. Indeed, although a Treg based cell therapy might control most of the autoimmune manifestations, the unknown *in vivo* life span of these cells, together with the possible need of a wider correction of the lymphoid compartment to resolve all the immunological defects, might call for a more stable and long-lasting HSC-targeted approach for IPEX.

Thus, we have tested in this work the effect of lentiviral vector (LV)-mediated constitutive expression of FOXP3 throughout hematopoiesis by transducing human CD34+ hematopoietic stem progenitors cells (HSPCs) and assessing their differentiation into an implemented NSG-based humanized mouse model.

## Results

### Modulation of the expression of FOXP3 affects HSPC *in vitro* maintenance and differentiation

**Figure 1:**
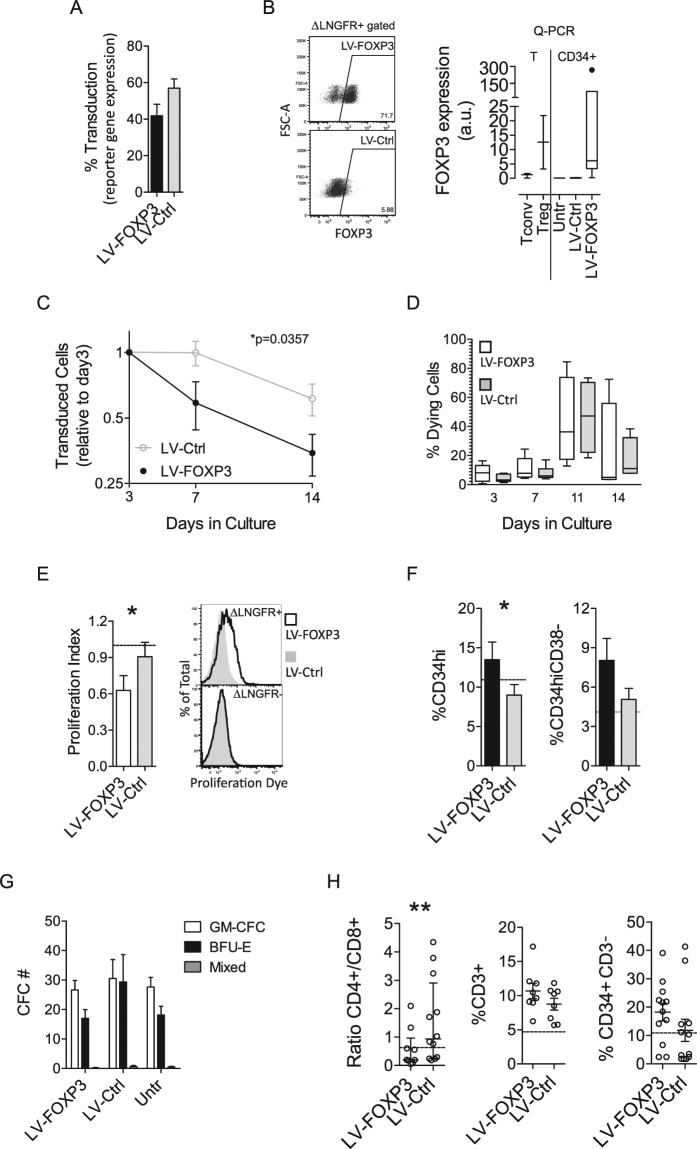
Constitutive expression of FOXP3 affects HSPC *in vitro* culture and differentiation. CB-derived CD34+ cells were transduced by LV expressing FOXP3 (LV-FOXP3) or a reporter gene (LV-Ctrl) and seeded either in liquid culture (**A**–**F**) or in semisolid medium (**G**) for 14 days, or in co-culture with OP9DL1 stromal cells for 21 days (**H**). (**D**–**F** and **H**) Analyses gated on transduced cell fractions. (**A**) Average transduction level by the indicated vectors, assessed at 4–7 days by reporter gene expression (n = 16) by flow cytometry. (**B**) FOXP3 expression, assessed by flow cytometry (left, representative plots) and Q-PCR (right), in CD34+ cells transduced by the indicated LV or untransduced (Untr) and in control T cells (Treg: CD4+CD25+ regulatory T cells; Tconv: CD4+CD25- conventional T cells) (n = 2–6). (**C**) Percentage of transduced cells, assessed by reporter gene expression in liquid culture by flow cytometry at the indicated time points after transduction; values are expressed as ratio to the percentage of transduced cells assessed at day 3; p value by two way ANOVA (n = 7). (**D**) Percentage of dying cells as assessed by AnnexinV or membrane integrity-based staining at 3, 7, 11 and 14 days after transduction. (**E**) Proliferation assessed 7 days after transduction by proliferation dye dilution and Ki67 staining. Left, proliferation index calculated as ratio of the percentage of proliferating cells in the indicated sample and the percentage of proliferating cells in the relative untransduced control. Right, representative flow cytometry histogram of proliferation dye dilution in ΔLNGFR + and - fractions. (**F**) Percentage of CD34high and CD34hiCD38- cells assessed 14 days after transduction. (**E**,**F**) Average values for untransduced cells are shown by dotted line. *p < 0.05 by one tail Wilcoxon matched pairs test (n = 6–9). (**G**) Average Colony Forming Cell numbers (CFC#) assessed in methylcellulose-based medium 14 days after plating of CD34+ cells transduced as indicated (n = 6); GM-CFC: granulocyte monocyte-CFC, BFU-E: burst forming unit-erythroid colonies. (**H**) Ratio of CD4+ and CD8+ percentages (left), percentage of CD3+ (middle) and percentage of CD34+ CD3- cells (right) assessed by flow cytometry after 21 days of OP9-ΔL1 co-culture. **p < 0.01 by two tail Wilcoxon matched pairs test (n = 12).

In order to study the impact of constitutive expression of FOXP3 on human hematopoiesis, we transduced cord blood-derived CD34+ HSPCs by LV-vectors expressing FOXP3 (LV-FOXP3) or a control gene (LV-Ctrl) and a reporter gene (either ΔLNGFR or GFP) (Fig. S1A). We obtained 42 ± 6.4% and 57 ± 5.1% reporter gene positive cells in LV-FOXP3 and LV-Ctrl transduced CD34+ cells, respectively (Fig. 1A). FOXP3 expression was well detectable at the protein level in most but not all ΔLNGFR+ LV-FOXP3 transduced CD34+ cells, likely reflecting a higher limit of detection for the intra-cytoplasmic FOXP3 staining compared to the membrane-bound ΔLNGFR. Indeed, FOXP3 RNA expression was comparable, if not higher, to the endogenous levels observed in Tregs, and indicated a very high FOXP3 expression *per* transduced cell when considering that only a fraction of the assessed CD34+ population was transduced and thus expressing FOXP3 (on average 40%, see Fig. 1A), while all Tregs homogenously express it (Fig. 1B) (see below for FOXP3 expression in ΔLNGFR sorted CD34+).

We performed a first set of *in vitro* experiments by culturing transduced HSPCs in liquid culture, clonogenic assays and T cell differentiation assays on OP9-ΔL1 stromal cells, and evaluated the effect of FOXP3 expression on proliferative and differentiation ability of these cells. We observed a significant reduction in the proportion of FOXP3 transduced cells in liquid culture during time, when compared to control transduced cells (transduced cells at day 14: 35 ± 7.5% vs 61 ± 10% of the relative transduction measured at day 3, LV-FOXP3 vs LV-Ctrl) (Fig. 1C), indicating a negative selection of LV-FOXP3 HSPCs. While FOXP3 expression did not significantly affect the mortality rate of cultured HSPC-derived cells at any of the time points assessed during the two weeks culture (Fig. 1D), it significantly reduced their proliferation rate (0.63 ± 0.12 vs 0.91 ± 0.12 proliferation rate relative to untransduced cells – set as 1 – 7 days post-transduction, LV-FOXP3 vs LV-Ctrl; Fig. 1E and Fig. S1B). Interestingly, we simultaneously observed increased maintenance of primitive CD34hi and CD34hiCD38- markers 14 days after transduction (14 ± 2.2% vs 9.0 ± 1.4% CD34hi, and 8.0 ± 1.7% vs 5.1 ± 0.84% CD34hiCD38-, LV-FOXP3 vs LV-Ctrl; Fig. 1F). The clonogenic potential of HSPCs was not affected by FOXP3 constitutive expression, as evaluated by semisolid culture (Fig. 1G). We then assessed the effect of FOXP3 expression on the ability of HSPCs to differentiate along the T cell lineage when co-cultured on OP9-ΔL1 stromal cells^14^. The percentage of CD3+ T cells arising from LV-FOXP3 and LV-Ctrl HSPCs was comparable (11 ± 1.1% vs 8.8 ± 0.86% CD34-CD3+ cells, LV-FOXP3 vs LV-Ctrl), while the CD4 +/CD8+ cell ratio was significantly reduced (0.20 range: 0.13–0.97 vs 0.93 range: 0.25–2.9, LV-FOXP3 vs LV-Ctrl CD4+ /CD8+ ratio), confirming the predominant role of FOXP3 in CD4+ T cells as compared to CD8+ (Fig. 1H). Similar to the data obtained in liquid culture, we observed a tendency for higher percentages of primitive cells, marked as CD34+CD3-, at the end of the co-culture in LV-FOXP3 when compared to control cells (18 ± 3.1% vs 12 ± 3.9% CD34+CD3- cells, LV-FOXP3 vs LV-Ctrl) (Fig. 1H). These data indicate that constitutive expression of FOXP3 might interfere with self-renewal/proliferation pathways in HSPCs and alter their differentiation into T cells.

### Constitutive expression of FOXP3 enhances repopulating ability of HSPCs

**Figure 2:**
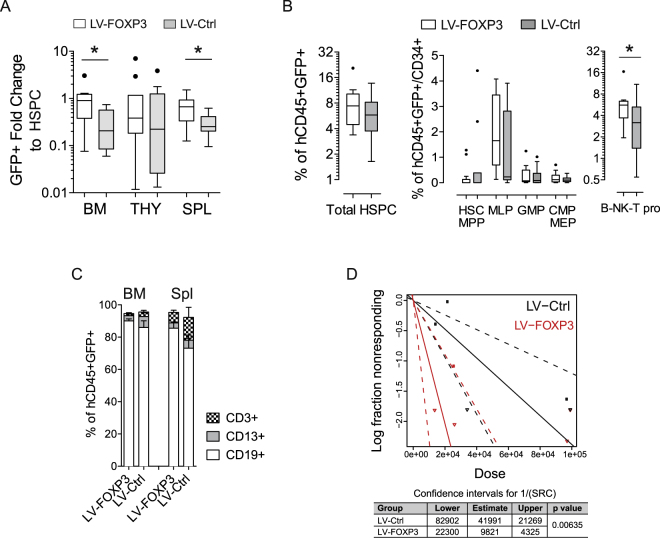
Constitutive expression of FOXP3 improves HSPC repopulation ability. CB-derived CD34+ cell transduced by LV expressing FOXP3 (LV-FOXP3) or a control gene (LV-Ctrl) were transplanted in the liver of sub-lethally irradiated NSG mice. Mice were analysed 15–18 weeks after transplant. (**A**) Fold change in percentage of GFP-expressing cells in the indicated tissues of the indicated experimental groups relative to the percentage of GFP-expressing cells assessed *in vitro* 3 days post-transduction. *p < 0.05 by two-tail Mann-Whitney test (n = 9–10). (**B**) Percentages of bone marrow progenitors in the engrafted GFP+ fraction assessed by flow cytometry. Left panel, total HSPC: CD34+ cells. Middle panel, HSC-MPP: hematopoietic stem cell-multipotent progenitors (CD34hiCD38-CD10-CD45RA-), MLP: multi-lymphoid progenitors (CD34hiCD38-CD10+ CD45RA+), GMP: granulocyte-monocyte progenitors (CD34+CD38+CD10-CD45RA+), CMP-MEP: common myeloid - megakaryocyte/erythrocyte progenitors (CD34+ CD38+ CD10-CD45RA-), B-NK-T pro: B-NK-T progenitors (CD34+CD38+CD10+CD45RA+). *p < 0.05 by two-tail Mann-Whitney test (n = 12). (**C**) Mature lineage distribution in the human CD45+GFP+ fraction of bone marrow (BM) and spleen (Spl) cells of the indicated experimental huMice. CD3+ = T cells, CD19+ = B cells, CD13+ = myeloid cells. (**D**) Extreme limiting dilution assay: NSG mice were transplanted with limiting dilution doses of LV-FOXP3 (red) or LV-Ctrl (black) CD34+ cells. Top, log-fraction plot of the limiting dilution model fitted to Table S1: logarithm of the fraction of non-repopulated mice (<1% CD45+GFP+ in the BM; Log fraction nonresponding) versus number of CD34+GFP+ cells transplanted per mouse (Dose); slope of the line: log-active cell fraction, dotted lines: 95% confidence interval, down-pointing triangle: cell dose with 0 non-repopulated mice. Bottom, confidence intervals for SRC frequency in the tested group (1/) and p value calculated by ELDA software (http://bioinf.wehi.edu.au/software/elda) are shown.

We transplanted equivalent starting cell doses of LV-FOXP3 and control transduced bulk CD34+ cells into the NSG-based humanized mouse model (huMice) we established and assessed human engraftment by flow cytometry at 15–18 weeks by the GFP+ (transduced) and GFP- (not transduced) fractions (Fig. S2A). On the basis of the results obtained *in vitro*, we then assessed a possible advantage in repopulating potential of FOXP3 transduced over the non-transduced HSPCs. If FOXP3 was conferring a competitive repopulating advantage to HSPCs, we would expect an *in vivo* enrichment of the GFP+ fraction in LV-FOXP3 when compared to LV-Ctrl transplanted huMice. We thus calculated for each huMouse *in vivo* GFP+ enrichment as fold change between the percentage of GFP+ cells in different engrafted tissues and the relative original percentages of GFP+ cells in infused HSPCs, and found that LV-FOXP3 transplanted huMice displayed a significantly higher fold change compared to controls in bone marrow and spleen (fold change 0.90 range: 0.38–1.2 vs 0.21 range: 0.08–0.57 in BM and 0.67 range: 0.33–0.93 vs 0.25 range: 0.20–0.41 in spleen, LV-FOXP3 vs LV-Ctrl) but not in the thymus (0.38 range: 0.18–1.2 vs 0.22 range: 0.03–1.2 fold change, LV-FOXP3 vs LV-Ctrl) (Fig. 2A). These results indicated a selective advantage of FOXP3-expressing HSPCs in repopulation, which was lost along the T cell lineage. Still, this analysis cannot discriminate between an effect of FOXP3 on the repopulating ability/lineage differentiation at the single cell level and an effect of FOXP3 on the number of repopulating cells in the HSPC population. To test the former hypothesis, we assessed the lineage differentiation and progenitor composition in periphery and bone marrow of LV-FOXP3 and LV-Ctrl transplanted huMice. We did not find major differences in LV-FOXP3 and LV-Ctrl huMice at the level of bone marrow composition and differentiation, beside the already mentioned trend towards reduced T cell differentiation, reflected in a significant accumulation of committed lymphoid progenitors in the bone marrow and lower percentages of CD3+ T cells in both bone marrow and periphery of LV-FOXP3 huMice (5.6 range: 3.7–6.4% vs 3.2 range: 1.4–5.3% CD34+CD38+CD10+CD45RA+ B-NK-T progenitors, LV-FOXP3 vs LV-Ctrl) (Fig. 2B,C). This result indicated that cell intrinsic repopulation and early differentiation was not affected by FOXP3 expression and suggested that FOXP3 rather affected the overall number of repopulating cells at the HSPC population level. In order to further test this latter hypothesis, we transplanted NSG mice with decreasing doses of transduced CD34+ cells and performed extreme limiting dilution analysis^15^. The fitted model showed highly different active cell fractions in the two groups, with a frequency of SCID Repopulating Cells (SRCs) in the LV-FOXP3 that was significantly higher (on average more than 4 folds) than the one in LV-Ctrl HSPCs (Fig. 2D, Table S1).

These data indicated that FOXP3 expression altered repopulating ability by increasing the numbers of repopulating cells in the HSPC population.

### Ectopic FOXP3 expression hampers *in vivo* T cell differentiation and response

**Figure 3:**
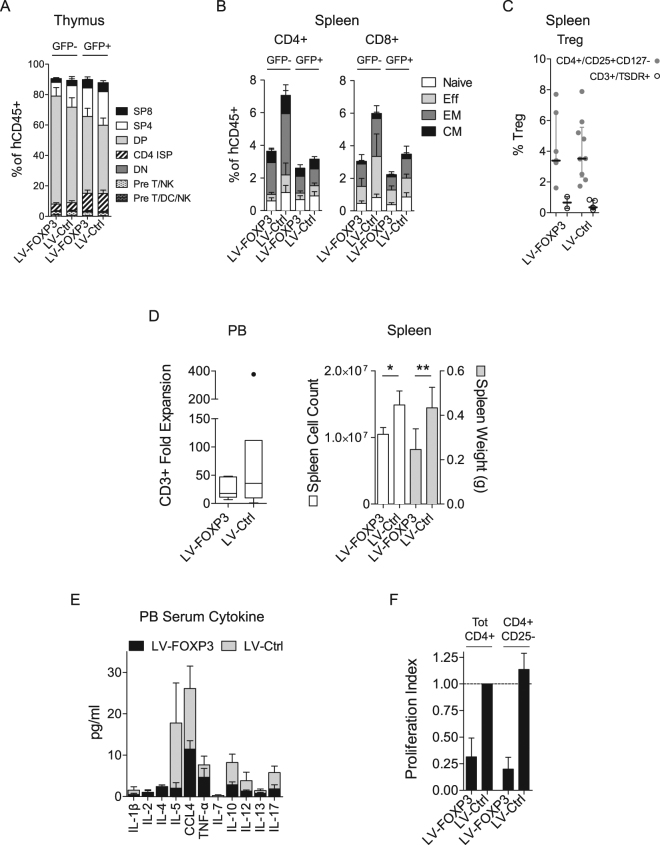
Constitutive expression of FOXP3 hampers T cell differentiation and response. CB-derived CD34+ cell transduced by LV expressing FOXP3 (LV-FOXP3) or a control gene (LV-Ctrl) were transplanted in the liver of sub-lethally irradiated NSG mice. 15–18 weeks after transplant mice were analyzed (**A**–**C**) or subjected to orthotopic injection of huIL7, huIL15 and huGM-CSF secreting tumor cells and analyzed 3 weeks after injection (**D**–**F**, n = 6). (**A**,**B**) Analysis performed on the GFP- (not carrying the vector) and GFP+ (carrying the vector) fractions, as indicated. (**A**) Percentages of thymocyte subpopulations assessed by flow cytometry; Pre T/DC/NK: myeloid/lymphoid precursors (CD1a-CD3-CD34+CD38lo), pre T/NK: lymphoid committed precursors (CD1a-CD3-CD34+CD38+), DN: double negative T progenitors (CD3-CD4-CD8-), CD4 ISP: CD4+ immature single positive T progenitors (CD3-CD4+CD8-), DP: double positive T progenitors (CD4+CD8+), SP4: single positive CD4 mature T cells (CD3+CD4+CD8-), SP8: single positive CD8 mature T cells (CD3+CD4-CD8+) (n = 13). (**B**) Percentages of splenic CD4+ and CD8+ T cell subpopulations; Naïve: CD62L+CD45RA+ ; TEMRA, terminally differentiated effector memory RA+ : CD62L-CD45RA+ ; EM, effector memory: CD62L-CD45RA-; CM, central memory: CD62L+CD45RA- (n = 10). (**C**) Percentage of splenic regulatory T cells (Treg), assessed by flow cytometry (filled circles) and by Q-PCR based analysis of TSDR (empty circles) (n = 2–9). (**D**) Left, CD3+ Fold expansion = ratio between absolute numbers of PB CD3+ T cells at sacrifice and absolute numbers of PB CD3+ T cells at day of tumor injection. Right, spleen cell count (white bars) and weight (grey bars). (**E**) Peripheral blood serum cytokines tested by multiplex ELISA in LV-FOXP3 (black bars) and LV-Ctrl (grey bars) huMice. (**F**) *In vitro* proliferation of sorted CD4+ and CD4+CD25- splenic cells upon anti-CD3/28/2 bead stimulus. Proliferation index is calculated as ratio between the percentage of proliferating cells in the depicted population and percentage of proliferating cells in tot CD4+ LV-Ctrl (dashed line) (n = 3). *p < 0.05 and **p < 0.01 by one-tail Mann Whitney test.

We then analysed the T cell compartment and observed an overall reduction in the T cell differentiation in LV-FOXP3 huMice compared to controls, with decreased percentages of mature thymocytes in both the vector carrying (GFP+) and not-carrying (GFP-) fractions (Fig. 3A). Accordingly, LV-FOXP3 huMice showed decreased percentages of peripheral CD4+ and CD8+ T cells compared to controls, while Treg frequencies were comparable (Fig. 3B,C). In order to verify if constitutive expression of FOXP3 affected the ability of T cells to respond to external cues, LV-FOXP3 and Ctrl huMice were orthotopically injected with breast cancer tumor cells MDA expressing human IL7, IL15 and GM-CSF, which has been previously shown to induce significant human T cells expansion in huMice^16^. By using this method, we tested *in vivo* T cell response to cytokines and *ex vivo* response to polyclonal stimulus by the *in vivo* expanded T cells. Three weeks post-implant, LV-FOXP3 huMice showed reduced peripheral T cell expansion (18 range: 11–47 vs 36 range: 9.9–111 folds increase in %CD3+, LV-FOXP3 vs LV-Ctrl) and smaller spleens (on average 52% and 74% of LV-Ctrl as cellularity and weight, respectively), as compared to Ctrl mice (Fig. 3D). Accordingly, when we assessed the presence of human cytokines in the serum of challenged huMice, we found inflammatory cytokines such as IL1β, IL5, CCL4, IL10 and IL17 significantly reduced in LV-FOXP3 compared to LV-Ctrl huMice (Fig. 3E). When we purified CD4 + cells from the spleen of challenged mice and stimulated them *in vitro* through the TCR, we observed reduced proliferation of LV-FOXP3 cells compared to Ctrl cells (0.31 ± 0.18 fold the proliferation index of LV-Ctrl on average) (Fig. 3F). Moreover, while the Ctrl CD4+ cells depleted of the Treg fraction (CD4+CD25−) proliferated more than total CD4+ cells as expected, CD4+CD25- LV-FOXP3 cells were still hypo-proliferative (0.20 ± 0.11 vs 1.1 ± 0.15 proliferation index, LV-FOXP3 vs LV-Ctrl) (Fig. 3F), indicating that FOXP3 overexpression intrinsically affected effector T cell proliferative capacity. Overall, these findings indicated that constitutive expression of FOXP3 in the hematopoietic system altered HSPC biology and reduced T cell differentiation and function.

### FOXP3 expression affects the expression of key genes in HSPC

**Figure 4:**
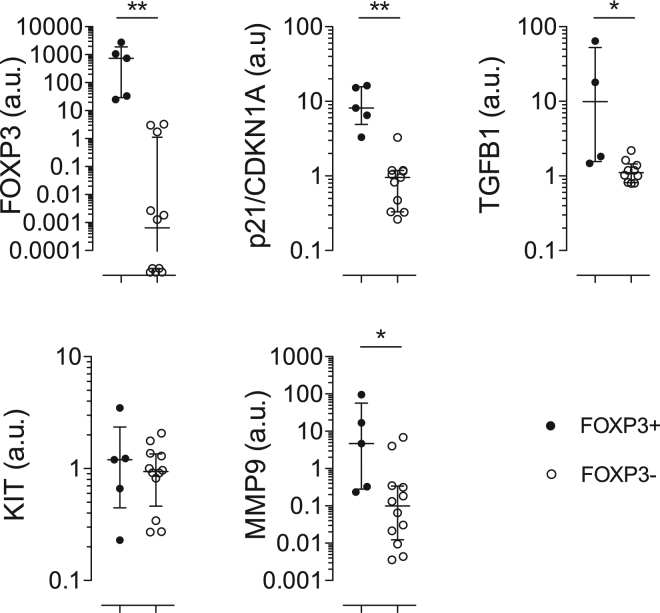
FOXP3 expression affects the expression of key genes in HSPC. dd-PCR analysis on CB-derived CD34+ cell transduced by LV expressing FOXP3 or a control gene and magnetically sorted in NGFR+ and NGFR- fractions. FOXP3+ (filled circles): NGFR+ fraction of LV-FOXP3 CD34+ ; FOXP3- (empty circles): NGFR- fraction of LV-FOXP3 cells, and both NGFR+ and NGFR- fractions of the LV-Ctrl cells. Values are expressed as arbitrary units: number of molecules/microliter of tested gene normalized on number of molecules/microliter of HPRT or TBP and relative to the average level of the FOXP3- group (set at 1). *p < 0.05 and **p < 0.01 by two tail Mann-Whitney test (n = 4–12).

In order to assess the molecular pathways affected by constitutive FOXP3 expression and underlying the observed outcome in HSPCs, we transduced CB CD34+ cells by LV-FOXP3 or LV-Ctrl, magnetically purified NGFR+ and NGFR- populations and assessed the expression of genes involved in maintenance of HSC primitive features and/or in controlling HSC niche. In particular, we assessed by dd-PCR analysis the tumor suppressor/cell cycle regulator p21/CDKN1A and the Transforming Growth Factor β1 (TGFB1) and found them significantly upregulated when FOXP3 was overexpressed compared to control HSPCs (FOXP3: 741 range: 29–1903 vs 0.001 range 0.00–1.3; p21/CDKN1A: 8.1 range: 4.9–16 vs 0.95 range: 0.33–1.2; TGFB1: 9.9 range: 1.6–53 vs 1.1 range: 0.81–1.4 arbitrary units FOXP3+ vs FOXP3-) (Fig. 4). This held true also for the gene encoding for the Matrix Metallopeptidase 9 (MMP9: 4.7 range: 0.28–56 vs 0.10 range: 0.01–0.34 arbitrary units FOXP3+ vs FOXP3-) (Fig. 4). KIT gene, encoding for the stem cell factor receptor, was not affected by FOXP3 expression (Fig. 4). Given the very well described role of p21 and TGFβ1 in controlling human HSC quiescence and self-renewal^17,18^, these data indicate that constitutive FOXP3 expression affects the main molecular pathways regulating HSC biology.

## Discussion

HSPCs do not physiologically express FOXP3. However, by studying in this work the effect of constitutive FOXP3 expression in human HSPCs during their hematopoietic differentiation in huMice, we demonstrate that FOXP3 expression, unexpectedly, preserves HSC features by increasing quiescence and repopulating ability. Constitutive FOXP3 expression resulted in reduced *in vitro* proliferation and a prominent increase in the frequency of SRCs in the HSPC population, and upregulated p21/CDKN1A and TGFB1, well known factors controlling quiescence and self-renewal of HSC^17,18^, and MMP9 genes. Interestingly, both p21/CDKN1A and TGFB1 have been associated to FOXP3 activity in different cell types. FOXP3 has been shown to directly bind to the first intron of the CDKN1A gene and increase its expression^19^, and TGFβ1 has been described by several studies as mediator of the immune suppressive ability exerted by Treg cells^20^. Therefore, our data might call for a direct effect of FOXP3 on p21/CDKN1A and TGFB1 expression also in HSPCs and suggest that they might be the molecular basis of the observed phenotype. Furthermore, a role for MMP9 protein, which belongs to a family of proteolytic enzymes involved in the degradation of the extracellular matrix and associated to tissue remodelling, has been proposed in HSC mobilization, though never fully proven in the human system^21^. Its expression is directly controlled by TGFB1 signaling^22^ and its proteolitic activity is required to activate latent TGFB1 itself^23^. MMP9 might thus be involved in the remodelling of HSC niche.

Our data thus indicate that, by affecting the molecular pathways controlling the main primitive features of HSC, ectopic FOXP3 expression improves the maintenance of more primitive HSC in the bulk HSPC population. These findings might open new avenues to improve human HSPC transplantation and gene manipulation. Indeed, induction of transient FOXP3 expression, by nucleofection of either FOXP3 RNA or artificial transcriptional activators to induce activation of the endogenous FOXP3 gene, might be envisaged to improve maintenance of repopulating human HSC. This may address an open need for many clinical applications, such as transplantation of CB-derived HSPC or gene edited HSPC^24,25^. The effect of FOXP3 expression on the number of SRC is less pronounced than the one obtained with the molecules nowadays approaching the clinics for HSPC expansion, such as SR1 and UM171 (4 fold for FOXP3 versus 17 and 13 fold for SR1 and UM171, respectively). Still, both approved compounds need a prolonged *in vitro* culture of HSPCs to be effective, as shown by the significant drop in the efficacy of SR1 when used for 12 instead of 21 days^26,27^. Instead, at least at the level of gene expression, FOXP3 effect is evident already at 5 days post-transduction. Even if only further experiment will clarify if transient expression will be as efficacious as constitutive in terms of SRC increase, we believe that the reduced culture time needed for ectopic FOXP3 expression might be an advantage compared to state-of-the-art compounds for the expansion of HSPCs, as it might reduce the risk of detrimental effects on HSC biology.

Ectopic FOXP3 expression as tool to specifically modulate HSPC features needs to be transient, since our data also show that, when FOXP3 expression persists, it impairs T cell differentiation and TCR-dependent/independent responses. These findings in the human system are consistent with the data obtained in FOXP3 transgenic mice^28^. Nevertheless, it is interesting to note that also GFP- cells (not carrying the vector and thus not expressing FOXP3) showed a similar, if not more pronounced, phenotype when compared to GFP+ (carrying the vector and expressing FOXP3) cells in huMice transplanted with LV-FOXP3 CD34+. This suggests that the fraction of FOXP3-expressing T cells affect the whole T cell compartment, possibly by altering the cytokine milieu. Consistently, LV-FOXP3 huMice displayed significantly reduced pro-inflammatory cytokines in the serum upon *in vivo* stimulation. These results clearly indicate that an HSC-based gene therapy approach for IPEX disease is not feasible by gene addiction but rather needs FOXP3 gene replacement preserving endogenous regulation of expression.

In conclusion, we discovered here a previously undescribed effect of FOXP3 on the HSPC compartment, which may unveil new pathways controlling HSC biology and could be exploited for clinical applications. Moreover, this work formally proves the need of maintaining endogenous FOXP3 gene regulation, such as by gene editing approaches or the use of lentiviral vectors containing endogenous FOXP3 regulatory regions, in HSC-based gene therapy for IPEX disease.

## Methods

### Cells and gene modification

Human cord blood HSPCs CD34+ were either purchased (Lonza) or purified by magnetic positive selection (Miltenyi Biotec) from healthy donors after informed consent approved by San Raffaele Ethics Committee and accordingly to Helsinki declaration. HSPCs were seeded and transduced in StemSpan medium (StemCell Technologies) supplemented with rhSCF, rhTPO, rhIL6 and rhFlt3-L (Peprotech) with 2 × 10^8^ TU/ml (MOI 200) LV transducing units, as previously described^29^, after 24hrs of prestimulation. Self-inactivating lentiviral vector constructs (Fig. S1A): bidirectional ΔLNGFR.CMVtata.EF1α.FOXP3.Wpre (LV-FOXP3) and ΔLNGFR.CMVtata.EF1α.nocDNA (LV-Ctrl) were used for *in vitro* experiments (Figs 1C–H and 4); bicistronic PGK.ΔLNGFRiresGFP.Wpre (LV-Ctrl) and PGK.FOXP3iresGFP.Wpre (LV-FOXP3), generated by cloning the FOXP3 coding sequence instead of the ΔLNGFR sequence into PGK.ΔLNGFRiresGFP.Wpre^30,31^, were used for *in vivo* experiments (Figs 2 and 3). Transduction, as assessed by reporter gene expression by flow cytometry, and expression, as assessed by Q-PCR on FOXP3 cDNA, efficiencies were comparable between the two sets of vectors and are shown as pooled in Fig. 1A,B.

### Humanized mouse model

2–5 days old NSG (NOD.Cg-Prkdc^scid^ Il2rg^tm1^WjI/SzJ, JAX mouse strain) mice were sublethally irradiated (1.5 cGy) and injected intrahepatically with 10^5^ CD34+ 5–7 hours later. CD34+ cells were counted at time of seeding, before transduction, and control and test mice were transplanted with equivalent starting cell doses. Mice were kept in sterile conditions in ventilated cages in SPF animal house and received irradiated food and, for 1 month after irradiation, Gentamycin (0.3mg/ml) in the drinking water. Animals were sacrificed at 15–18 weeks, unless otherwise specified, and peripheral blood, bone marrow, thymus and spleen were harvested. Mice showing < 1% hCD45+GFP+ cells in the bone marrow were excluded from further analyses. Cytokine producing tumor xenograft was performed and analysed as previously described^16^. Briefly, 4 × 10^6^ mammary carcinoma MDA3.1 cells (9/10 MDA-MB231 and 1/10 MDA-MB231 stably expressing human IL7, IL15 and GM-CSF) were implanted orthotopically in the mammary fat pad of huMice 15 weeks post CD34+ injection. Mouse health was monitored for three weeks, at the end of which huMice were sacrificed and lymphoid organs (see above) harvested. All protocols involving animals followed the Decreto Legislativo number 116 dated January 27th 1992 from the Italian Parliament and have been evaluated and approved by the San Raffaele Ethics Committee (IACUC protocol 488 and 632) as following the 3R principles.

### *In vitro* assays

Liquid culture of CD34+ cells and CFC assay were previously described^32^. Briefly, CD34+ were cultured in StemSpan or IMDM 5% fetal bovine serum medium (StemCell Technologies) in presence of rhSCF (100ng/ml), rhIL6 (20ng/ml), rhFlt3-L (100ng/ml) and rhTPO (20ng/ml) (all from Peprotech) for liquid culture, while for CFC assay were plated at 10^3^ cells/ml in semi-solid medium (MethoCult H4434 Classic - StemCell technologies) and scored by microscope 14 days later. Proliferation and mortality of liquid cultures were assessed at 3, 7, 11 and 14 days post-transduction. OP9-ΔL1 stromal cell line was kindly provided by I. Schmutz and M. Cavazzana and co-culture was performed as described for 21 days^14^. *In vitro* proliferation of T cells was assessed by cell proliferation dye efluor670 (eBioscience) staining of total splenocytes or magnetically purified CD4+ and CD4+CD25- cells, where indicated, upon stimulation by antiCD3/CD28/CD2 beads (Treg suppression inspector beads – Miltenyi Biotec) following manufacturer’s instructions.

### Flow Cytometry

Transduction level was assessed either by direct GFP measurement or anti-LNGFR-PE or -APC staining (anti-CD271, Miltenyi Biotech). Proliferation and mortality of HSP-derived cultured cells were assessed by Ki67-PE or CFSE-based dies, and AnnexinV or live/dead staining kits (all from BD biosciences). CD34+/OP9-ΔL1 co-culture was harvested and filtered by 50μ cell strainer; the resulting hematopoietic cells were stained by anti-human CD34-PECy7, CD3-PerCPCy5.5, CD4-PB, CD8-APCH7, CD25-APC, CD127-PercPCy5.5, FOXP3-Alexa488 and LNGFR-PE. For the characterization of human hematopoietic cells derived from mouse tissues, single cell suspensions were obtained by physical disruption of lymphoid organs and lysis of red blood cells by ammonium chloride solution (StemCell Tecnologies). The following antibody panels were used: anti-human CD45-V500, CD34-PECy7, CD10-APC, CD38-PerCP, CD45RA-PB (bone marrow progenitors); anti-human CD45-PB, CD1a-PE, CD3-V500, CD34-PECy7, CD38-PerCPCy5.5, CD4-APC, CD8-APCH7 (thymocytes); CD45-V500, CD3-PerCPCy5.5 or -APC, CD4-PECy7, CD8-APCH7, CD62L-PE, CD45RA-PB (T cells); CD45-V500, CD4-PECy7, CD8-APCH7, CD25-PB, CD127-PerCPCy5.5, FOXP3-Alexa647, (Treg) (intracellular staining by Fix/Perm kit, following manufacturer’s instruction - eBiosciences). *In vitro* T cell proliferation from splenic cells was assessed 6 days after seeding by direct analysis of the proliferation dye dilution and staining by anti-human CD3-PerCPCy5.5, CD4-PECy7 and CD8-PE.

Antibodies purchased from BD Biosciences, eBiosciences and BioLegend. All acquisitions performed by FACSCanto II (Beckman Coulter) and analyzed by FlowJo Software.

### TSDR sequencing

Genomic DNA was extracted by Qiagen DNeasy kit from total splenocytes obtained from huMice. Treg-Specific Demethylated Region (TSDR) was assessed and quantified by Epiontis Biotech normalized to hCD3 locus, as described^33^.

### Gene expression analysis

RNA was extracted from transduced CD34+, after magnetic purification of NGFR+ and NGFR- fractions, 5 days after transduction by ReliaPrep RNA Miniprep kit (Promega), following manufacturer’s instructions. RNA was retrotranscribed by High Capacity cDNA kit (LifeTechnologies), and the level of expression of specific genes was assessed on 2–5ng or 20ng of RNA equivalent by digital droplet (dd)-PCR (QX200 Bio-Rad) or Q-PCR (Applied Biosystems 7900HT Fast Real-Time PCR), respectively, using specific primers (provided upon request) and normalized on HPRT and/or TBP expression. Analyses were performed by QuantaSoft (dd-PCR) or SDS (Q-PCR) softwares.

### Statistical methods

Values are expressed as mean ± SEM in column bar, and median and interquartile range in box / scatter plot graphs. Data were analysed by non-parametric tests based on ranks. The specific test used for each experiment is described in the figure legend. Groups were considered different at p < 0.05, only significant p values are reported. Extreme limiting dilution assay was performed by ELDA software (http://bioinf.wehi.edu.au/software/elda)^15^.

### Data availability

The datasets generated during and/or analysed during the current study are available from the corresponding author on reasonable request.

## Electronic supplementary material

Supplementary Information
