# Supplementary material for: Ectopic FOXP3 Expression Preserves Primitive Features Of Human Hematopoietic Stem Cells While Impairing Functional T Cell Differentiation
Source: Sci Rep. 2017 Nov 17;7:15820. doi: 10.1038/s41598-017-15689-8 (PMC5693945; doi:10.1038/s41598-017-15689-8)
Supplement: Supplementary file 1 — Supplementary Information [file 41598_2017_15689_MOESM1_ESM.pdf]

SUPPLEMENTARY INFORMATION

# Ectopic FOXP3 Expression Preserves Primitive Features Of Human Hematopoietic Stem Cells While Impairing Functional T Cell Differentiation

F.R. Santoni de Sio, L. Passerini, M. M. Valente, F. Russo, L. Naldini, M.G. Roncarolo, R. Bacchetta

Figure S1

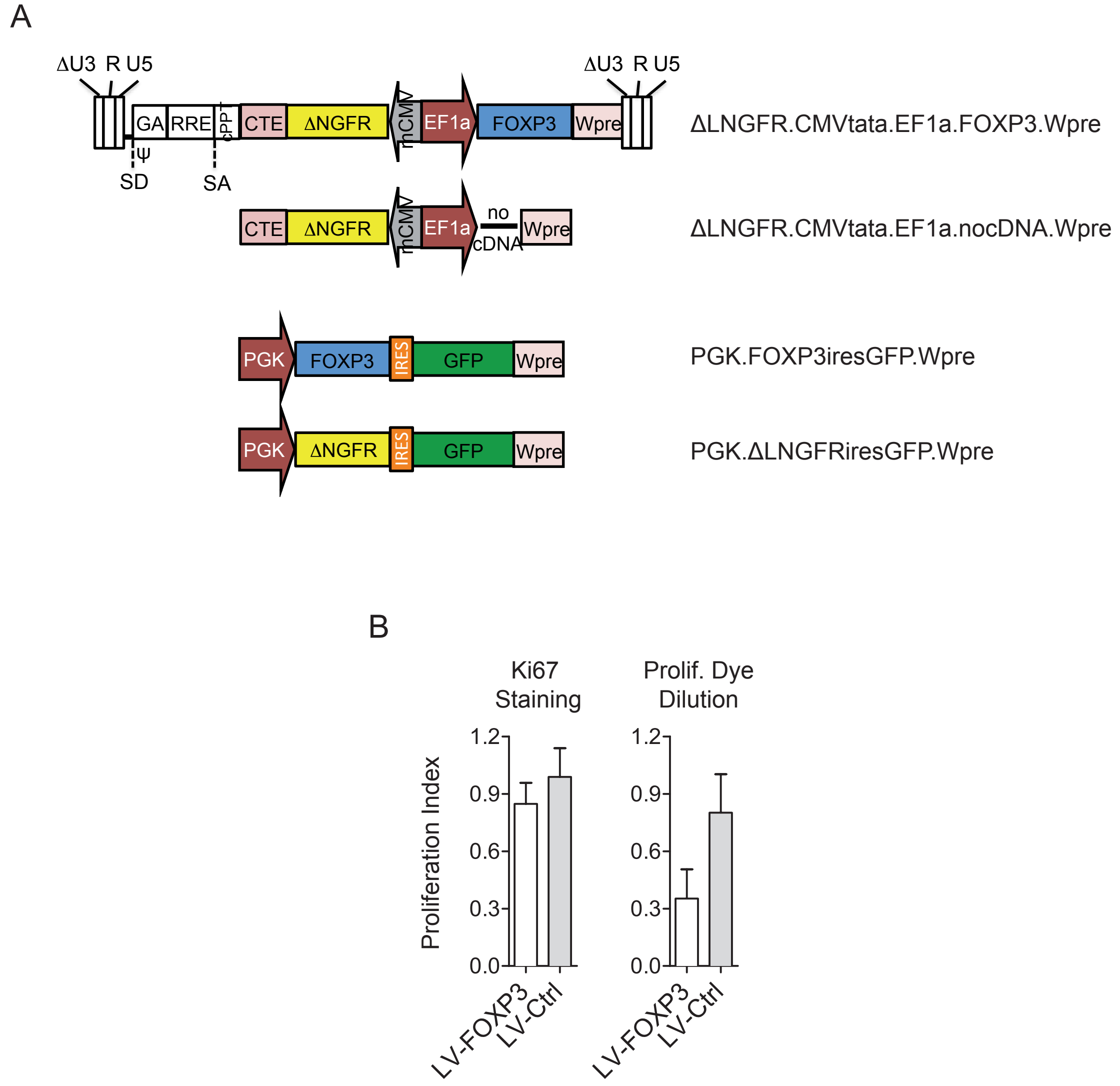


A) Schematic of the self-inactivating lentiviral vector constructs used in this work. Bidirectional (top) and bicistronic (bottom) vectors encoding FOXP3 and a reporter gene were used. EF1a: Elongation Factor 1a promoter; mCMV: minimal human Citomegalovirus promoter, ΔLNGFR: truncated Light Nerve Growth Factor Receptor reporter gene, Wpre: woodchuck hepatitis virus post-transcription regulatory element, CTE: Constitutive Transport Element from the Mason-Pfizer monkey virus, PGK: human phosphoglycerate kinase promoter, IRES: Intra Ribosome Entry Site, GFP: Green Fluorescent Protein reporter gene. In the top panel the proviral integrated form of the vector is also provided, including the HIV-derived regions: ΔU3, R and U5, LTR regions with deletion in U3, SD and SA, splice donor and acceptor site; ψ,encapsidation signal including the 5' portion of the gag gene (GA), RRE, Rev-response element, cPPT, central polypurine tract.
B) Proliferation assessed 7 days after transduction by Ki67 staining (left) and proliferation dye dilution (right). Proliferation index calculated as ratio of the percentage of proliferating cells in the indicated sample and the percentage of proliferating cells in the relative untransduced control.

Figure S2

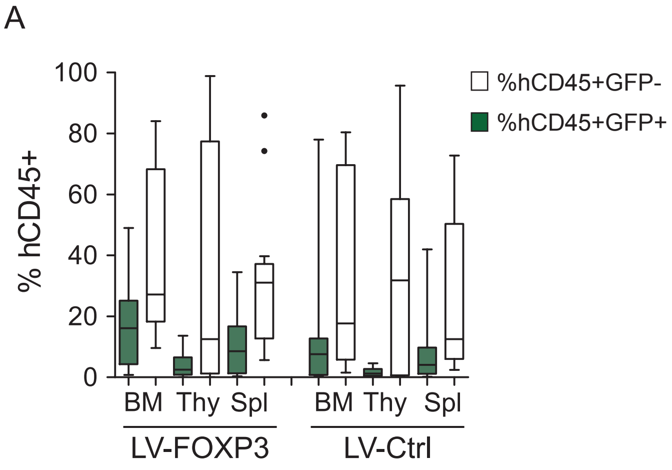


CB-derived CD34+ cell transduced by LV expressing FOXP3 (LV-FOXP3) or a control gene (LV-Ctrl) were transplanted in the liver of sub-lethally irradiated NSG mice. Mice were analyzed 15-18 weeks after transplant.
A) Median hCD45+GFP+ (green bars) and hCD45+GFP- (white bars) percentages in bone marrow (BM), thymus (Thy) and spleen (Spl).

**Table S1. Extreme limiting dilution analysis.**

| Number of CD34+GFP+ cells transplanted (Dose)[a] | Transplanted mice (Tested) | Repopulated mice (Response)[b] | Group |
|---|---|---|---|
| 99000 | 3 | 3 | LV-FOXP3 |
| 97000 | 5 | 5 | |
| 26000 | 4 | 4 | |
| 25721 | 3 | 2 | |
| 13500 | 3 | 3 | |
| 99000 | 3 | 3 | LV-Ctrl |
| 97000 | 5 | 4 | |
| 34000 | 3 | 3 | |
| 21785 | 3 | 0 | |
| 14250 | 3 | 1 | |

[a] Calculated on the basis of the percentage of transduction in the transplanted cell population. 100000 total cells per mouse were transplanted in all experiments.
[b] CD45+GFP+≥1% in the bone marrow.
